# Harmonization of PET image reconstruction parameters in simultaneous PET/MRI

**DOI:** 10.1186/s40658-021-00416-0

**Published:** 2021-11-05

**Authors:** Richard Laforest, Mehdi Khalighi, Yutaka Natsuaki, Abhejit Rajagopal, Dharshan Chandramohan, Darrin Byrd, Hongyu An, Peder Larson, Sara St. James, John J. Sunderland, Paul E. Kinahan, Thomas A. Hope

**Affiliations:** 1 Mallinckrodt Institute of Radiology, Washington University, St. Louis, MO USA; 2 Department of Radiology, Stanford University, Stanford, CA USA; 3 Department of Radiation Oncology, University of California San Francisco, San Francisco, CA USA; 4 Department of Radiology and Biomedical Imaging, University of California San Francisco, San Francisco, CA USA; 5 University of Iowa, Iowa City, IA USA; 6 University of Washington, Seattle, WA USA

**Keywords:** Image reconstruction, Harmonization, PET/MRI, Phantom

## Abstract

**Objective:**

Simultaneous PET/MRIs vary in their quantitative PET performance due to inherent differences in the physical systems and differences in the image reconstruction implementation. This variability in quantitative accuracy confounds the ability to meaningfully combine and compare data across scanners. In this work, we define image reconstruction parameters that lead to comparable contrast recovery curves across simultaneous PET/MRI systems.

**Method:**

The NEMA NU-2 image quality phantom was imaged on one GE Signa and on one Siemens mMR PET/MRI scanner. The phantom was imaged at 9.7:1 contrast with standard spheres (diameter 10, 13, 17, 22, 28, 37 mm) and with custom spheres (diameter: 8.5, 11.5, 15, 25, 32.5, 44 mm) using a standardized methodology. Analysis was performed on a 30 min listmode data acquisition and on 6 realizations of 5 min from the listmode data. Images were reconstructed with the manufacturer provided iterative image reconstruction algorithms with and without point spread function (PSF) modeling. For both scanners, a post-reconstruction Gaussian filter of 3–7 mm in steps of 1 mm was applied. Attenuation correction was provided from a scaled computed tomography (CT) image of the phantom registered to the MR-based attenuation images and verified to align on the non-attenuation corrected PET images. For each of these image reconstruction parameter sets, contrast recovery coefficients (CRCs) were determined for the SUV_mean_, SUV_max_ and SUV_peak_ for each sphere. A hybrid metric combining the root-mean-squared discrepancy (RMSD) and the absolute CRC values was used to simultaneously optimize for best match in CRC between the two scanners while simultaneously weighting toward higher resolution reconstructions. The image reconstruction parameter set was identified as the best candidate reconstruction for each vendor for harmonized PET image reconstruction.

**Results:**

The range of clinically relevant image reconstruction parameters demonstrated widely different quantitative performance across cameras. The best match of CRC curves was obtained at the lowest RMSD values with: for CRC_mean_, 2 iterations-7 mm filter on the GE Signa and 4 iterations-6 mm filter on the Siemens mMR, for CRC_max_, 4 iterations-6 mm filter on the GE Signa, 4 iterations-5 mm filter on the Siemens mMR and for CRC_peak_, 4 iterations-7 mm filter with PSF on the GE Signa and 4 iterations-7 mm filter on the Siemens mMR. Over all reconstructions, the RMSD between CRCs was 1.8%, 3.6% and 2.9% for CRC mean, max and peak, respectively. The solution of 2 iterations-3 mm on the GE Signa and 4 iterations-3 mm on Siemens mMR, both with PSF, led to simultaneous harmonization and with high CRC and low RMSD for CRC mean, max and peak with RMSD values of 2.8%, 5.8% and 3.2%, respectively.

**Conclusions:**

For two commercially available PET/MRI scanners, user-selectable parameters that control iterative updates, image smoothing and PSF modeling provide a range of contrast recovery curves that allow harmonization in harmonization strategies of optimal match in CRC or high CRC values. This work demonstrates that nearly identical CRC curves can be obtained on different commercially available scanners by selecting appropriate image reconstruction parameters.

**Supplementary Information:**

The online version contains supplementary material available at 10.1186/s40658-021-00416-0.

## Introduction

Positron emission tomography (PET) allows for the measurement of absolute activity concentration of radiotracers in vivo with high sensitivity and high accuracy. Repeatable and reproducible measurements of tracer uptake in terms of, for example, standardized uptake value (SUV), are essential for monitoring and quantifying tumor response to therapy or progression of disease. In the context of multicenter clinical trials where data are pooled from different sites with different makes and model of scanners, vastly different quantitative performance characteristics may exist, limiting the ability to draw meaningful conclusions from these trials. This is the case whether it is a trial limited to PET/MRI scanners exclusively, or where PET/MRI data are included along with PET/CT data.

Variability of SUV measurements in clinical PET has been described before [1, 2] and has been associated with three main causes: technical (including absolute scanner calibration, dose calibrator calibration, residual activity in the syringe after administration, clock synchronization), biological (including patient preparation, inherent physiologic variability which is also tracer dependent, patient movement) or physical (including acquisition and image reconstruction parameters, ROI placement, scanner design) [1]. To achieve reproducible measurements of activity by PET involves an adequate quality control program of the scanner to ensure operation with accurate calibration to minimize bias but also with standardization in patient preparation and imaging to minimize biological variability [3]. Physical variability can be minimized by ensuring that the acquisition parameters and image reconstruction parameters be chosen to minimize the difference in SUVs across scanners from different sites or manufacturers. In particular, attention has been given to the choice of image reconstruction parameters; sites cannot change the systems installed, but with careful choice of image reconstruction parameters, differences between sites may be minimized. Quantitative PET is substantially affected by the choice of image reconstruction parameters which may differ between institutions, and also by different scanner technology and differences in implementation of image reconstruction algorithms among vendors. Furthermore, the recent introduction of resolution modeling in the PET reconstruction has led to greater variability especially in terms of SUV_max_ in small tumors [4, 5]. Numerous efforts have been implemented to minimize the variability of clinical PET, mainly for ^18^F-FDG, such as RSNA-QIBA [6], SNMMI-CTN [7] and EANM [8, 9] in the context of PET/CT. These efforts aimed at proposing specifications and requirements in the patient preparation, injection and imaging in order to provide comparability and consistency for quantitative FDG-PET across scanners in oncology.

The aim of harmonization in PET is different from standardization. Standardization implies that sites use a uniform procedure with the goal to minimize variations, while harmonization aims at achieving comparable results across manufacturers or sites even though slightly different procedures are used. Both harmonization and standardization reduce variations, but harmonization encompasses standardization which is stricter, but in this work we chose to concentrate on harmonization strategies. Harmonization aims at achieving the same level of accuracy across the imaging system and thus aims at minimizing variations or determines limits in tolerable variation. Most commonly in PET/CT, this harmonization has been performed through the use of carefully tuned scanner model-specific post-reconstruction filtration [7, 10, 11]. The purpose of harmonization of image reconstruction parameters is thus different from evaluating the convergence properties and reproducibility of lesion contrast recovery coefficients and their dependence on the choice of parameters. The purpose of harmonization is to determine the image reconstruction parameters that minimize variations in resolution recovery (as opposed to achieving similar noise properties, for example) upon imaging a subject similarly prepared on different imaging systems. Image generation for PET from PET/MRI is functionally the same as for PET/CT. The main differences are the scanner geometry which typically utilizes a smaller ring diameter and longer axial field of view as compared to PET/CT and the use of MR-based attenuation correction techniques.

**Fig. 1:**
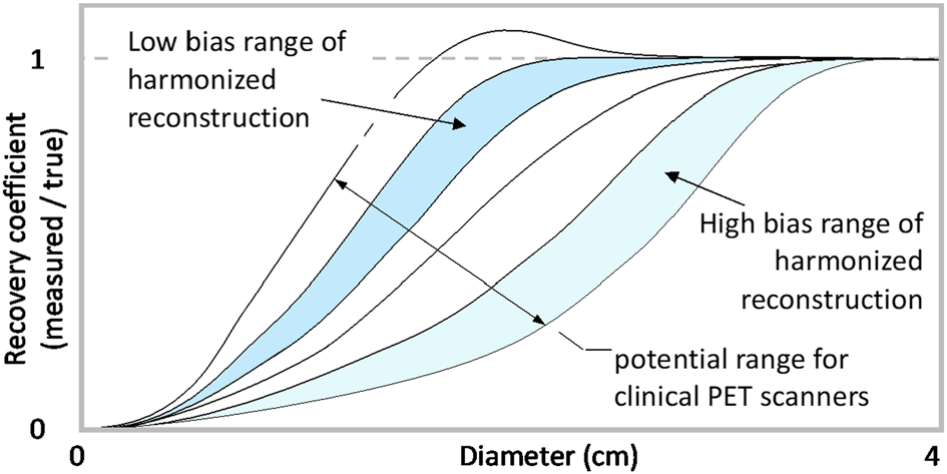
Conceptual illustration of quantitative harmonization for PET imaging using recovery coefficients. In this case, bias is the difference between the measured recovery coefficient and the ideal value of 1.0

The objective of quantitative harmonization differs from the objective of maximizing resolution or recovery coefficients. We define harmonization as having three components: The first is having measures of both bias and variance of image regions of interest (ROIs) of different sizes. Second is the minimization of the quantitative inaccuracy between ROIs on images from different systems. The quantitative inaccuracy can be expressed as bias, or the root-mean-square error (RMSE), or the coefficient of variation (COV), or combinations of these or other metrics. The third component is the desired performance envelope as conceptually indicated in Fig. 1. For example, a high-bias range may enable inclusion of scanners with lower resolution, while a low-bias range of performance may be more suitable for the study goals.

The focus of this study is low-bias quantitative harmonization for small lesions imaged on two different PET/MR systems. The method we have employed follows the procedure described in Makris et al. [11], Byrd et al. [12], and Sunderland et al. [7]. With our choice of metric, it is possible that the image noise will be different between the images from the two scanners as they have different sensitivities and image reconstruction algorithms. We note that achieving the same signal/noise ratio is not the objective of quantitative harmonization in this clinical setting.

Currently, in the USA two manufacturers (Siemens and General Electric) offer PET/MRI scanners, although a third vendor is poised to enter the US market (United Imaging). These two currently available PET/MRI scanners differ in the choice in scintillation crystal (size and material), overall detector geometry, photo-multiplication technology (avalanche photodiode vs silicon photomultiplier array) and in their image reconstruction algorithms. Although both vendors provide ordered-subset expectation–maximization algorithm (OSEM), the implementation of the algorithm varies between vendors and the performance of the algorithm is expected to differ due to the use of different number of subsets, number of iterations, data compression prior to reconstruction, implementation of the system matrix, crystal size and axial plane thickness as well as from the different implementations of the point spread function modeling. The aim of this study was to determine sets of changeable by the user and clinically relevant image reconstruction parameters on both systems that yield the optimal match in quantitative performance as a function of object size for tumor like objects. The study was performed on one scanner from each vendor with controlled phantom experiment to minimize errors in phantom filling and focus on isolating effects on accuracy of measurements from the specifics of scanner hardware and image reconstruction algorithms.

## Methods

PET imaging was performed using the Siemens Biograph mMR (Siemens Healthineers, Erlangen, GE) and with the General Electric Signa PET/MRI (General Electric, Wisconsin, USA). Scanner characteristics are compared in Table 1, and their performance evaluation was previously reported [13, 14]. Phantom imaging was performed using the NEMA IEC Phantom [15] (Data Spectrum Corp., North Carolina, USA) using the standard set of spheres (diameters: 10, 13, 17, 22, 28 and 38 mm) and a custom set of spheres (diameters: 8.5, 11, 15, 25, 32.5 and 44 mm). The second set of spheres contains intermediate sphere sizes and provides more data points for measurement and optimization. A standardized filling procedure was implemented aiming to achieve a 9.8:1 ratio between the spheres and the water background. The procedure followed EANM FDG PET PET/CT guidelines [16]; two doses of approximately 20 MBq of ^18^F were used. The first dose was diluted in 1000 mL of water, and the second dose was diluted into the approximately 9700 mL background water volume of the phantom chamber. The water volume of the phantom was determined by weight. The phantom was then centered in the PET field of view ensuring all six spheres are in the same imaging plane. On the Siemens scanner, this was ensured by placing the phantom on a foam cradle. PET data acquisitions were performed in listmode for 30 min. A two-point DIXON [17] (LavaFlex on GE Signa) attenuation scan was acquired but was only used to register a phantom CT attenuation template as described below.

**Table 1:** Compilation of representative scanner parameters

	Siemens Biograph mMR	General Electric Signa
Crystal size	4 × 4 × 20 mm^3^	4 × 5.3 × 25 mm^3^
Crystal material	LSO	LYSO
Light sensing device	Avalanche photodiode	Si-photomultiplier
Ring diameter	65.6 cm	66 cm
Field of view	59.4 cm × 25.6 cm	60 cm × 25 cm
PSF/TOF capable	Yes/no	Yes/yes
Sensitivity	15 cps/kBq	21 cps/kBq
Peak NECR	183.5 kcps @ 23.1 kBq/mL	210 kcps @ 23.1 kBq/mL
Spatial resolution at 1 cm	4.3/4.3 mm	4.3/5.34 mm
Volumetric spatial resolution at 10 cm	165 mm^3^	173 mm^3^

Images were reconstructed using the vendor provided image reconstruction software with a range of parameters encountered in the clinic for oncologic whole-body PET. Data from the GE Signa scanner were reconstructed offline with the GE’s *Duetto* PET reconstruction toolbox (v02.06) using 3D-OSEM (ordered-subset expectation–maximization) [18] algorithm with time of flight (TOF), at 2 and 4 iterations, 16 subsets, with and without point spread function (PSF) resolution modeling. On the Siemens mMR, images were reconstructed with *e7tools* (VE11P-SP2) using 3D-OSEM, 1–4 iterations, 21 subsets, with and without PSF resolution modeling. The number of subsets is set at 21 by the manufacturer on Siemens mMR. We set the number of subsets on the GE Signa at 16. The number of iterations on the GE Signa was selected from clinical use protocols for oncological PET. On the Siemens system, the number of iterations was selected to encompass the number of image updates (defined by the product of number of iterations times the number of subsets). As such, GE Signa images were reconstructed at 32 and 64 image updates, and the images on Siemens mMR were reconstructed with a range of 21–84 updates. Time of flight (TOF) was employed on the GE Signa as non-TOF PET reconstruction is not typically performed on systems allowing TOF. TOF benefits have been well documented to reduce image variance, thereby reducing signal to noise, improving convergence rate and reducing artifacts [19, 20], and are typically used whenever available. Siemens mMR does not have the TOF option available.

All reconstructions were repeated with a post-reconstruction Gaussian filter ranging from 3 to 7 mm. Images were reconstructed on a common voxel size of 2.34 mm in the transverse direction on a 256 matrix. GE Signa images were reconstructed on the native 2.78 mm slice thickness with standard axial filtering, while the Siemens mMR images were reconstructed on the native 2.027 mm slice thickness. Measured attenuation of the phantom by MRI methods such as DIXON or LavaFlex leads to inaccurate attenuation maps since the phantom material (water and plastic phantom wall) does not accurately mimic human tissue; water-like material will appear distorted, while plastics do not show at all [21]. The phantom also contained a 50-mm-diameter cylindrical plastic insert filled with polystyrene and water to mimic the lungs. Consequently, standard tissue segmentation algorithm will fail when applied to the reconstructed image of the phantom. To avoid these issues, attenuation correction of the NEMA phantom is provided by the manufacturers using a template stored in the system. A CT-based template phantom attenuation map was used on both scanners and registered to the TOF-NAC PET images via rigid registration. Accuracy of the registration of the template was further visually inspected and verified by inspection of the resulting mu-map over the non-attenuation corrected PET images (See Additional file 1: Fig. S1).

In a first analysis, images were reconstructed using the entire 30-min listmode data acquisition. In a second analysis, the 30-min listmode data were fragmented into 6 frames of approximately 5 min (277, 286, 295, 304, 314 and 324 s). The increasing frame duration ensured approximately equal number of collected events in each realization when accounting for radioactive decay. Contrast recovery coefficients (CRCs) as defined by Liow and Strother [22] were calculated using the scanner measured sphere activity concentration, the scanner measured background water activity concentration and the sphere and background water activity concentration calculated from the assayed activities, dilution data and decay corrected to scan time.

1 $${\text{CRC}} = \frac{{\left( {{\raise0.7ex\hbox{$S$} \!\mathord{\left/ {\vphantom {S B}}\right.\kern-\nulldelimiterspace} \!\lower0.7ex\hbox{$B$}}} \right)_{{{\text{measured}}}} - 1}}{{\left( {{\raise0.7ex\hbox{$S$} \!\mathord{\left/ {\vphantom {S B}}\right.\kern-\nulldelimiterspace} \!\lower0.7ex\hbox{$B$}}} \right)_{{{\text{theory}} }} - 1}}$$

All images were resampled to provide cubic voxels of approximately 1 mm^3^. Contrast recovery coefficients were computed using three methodologies: from the average sphere activity in a spherical volume of interest (VOI) drawn with diameter of the physical inner sphere diameter (CRC_mean_), from the maximum value in each sphere (CRC_max_) and from the peak value in each sphere defined as the average of a 1 cm^3^ VOI with highest value (CRC_peak_) within the physical sphere. The definition of SUV_peak_ of [23] was applied to define CRC_peak_. As defined, the CRC_peak_ may or may not include the hottest pixel with the sphere. Results of the two sets of spheres were combined to provide CRC curves against the 12 sphere sizes ranging from 8.5 to 44 mm. The background water activity was measured as the average over two 50-mm-diameter circular regions of interest localized in five adjacent image planes of the phantom without spheres. These ROIs were used to define the image roughness which is a measure of the apparent noise [4]. The average coefficient of variation (COV) over those ten regions defines the image roughness (IR) and is calculated by

2 $${\text{IR}}\left( \% \right) = 100*\frac{1}{10}\mathop \sum \limits_{k} \frac{{{\text{STD}}_{k} }}{{{\text{Mean}}_{k} }}$$

where STD_*k*_ is the standard deviation of the pixel intensity, the Mean_*k*_ is the average in region *k* and 10 the number of ROIs drawn in the background area. The root-mean-squared discrepancy (RMSD) for all 800 image reconstruction parameter combination pairs was then calculated.

3 $${\text{RMSD}} = 100*\sqrt {\frac{1}{12}\mathop \sum \limits_{{{\text{spheres}}}} \left[ {{\text{CRC}}_{{{\text{Signa}}}}^{i} - {\text{CRC}}_{{{\text{mMR}}}}^{j} } \right]^{2} }$$

where *i,j* are the image reconstruction index from the GE Signa and Siemens mMR and the summation extends over all 12 spheres. The optimized PET imaged reconstruction parameter set (#iterations, filter width or use of PSF or not) was determined by selecting parameters that minimized the following hybrid metric:

4 $$F\left( {\text{iterations, filter, PSF/IR}} \right) = \arg \min \left\{ {{\raise0.7ex\hbox{${{\text{RMSD}}}$} \!\mathord{\left/ {\vphantom {{{\text{RMSD}}} {a }}}\right.\kern-\nulldelimiterspace} \!\lower0.7ex\hbox{${a }$}} - \frac{\beta }{12}\sum {\text{CRC}}_{{{\text{mMR}}}}^{i} {\text{CRC}}_{{{\text{Signa}}}}^{i} } \right\}$$

RMSD was determined for CRC_mean_, CRC_max_ and CRC_peak_, and the constant *a* is chosen to normalize RMSD so that $\sum {\text{CRC}}_{{{\text{mMR}}}}^{i} {\text{CRC}}_{{{\text{Signa}}}}^{i}$ (referred as the sum CRC product) to be on the same scale (*a* was set equal to 20 throughout) and was set to the maximum value of RMSD observed in our analysis over the parameter set. The sum of the product of the CRC coefficients will reach a maximum value for images with higher spatial resolution and thus with less smoothing. The constant *β* was used to select solutions for either lowest RMSD (*β* = 0) or maximize CRC (*β* ~  = 1). An intermediate value of *β* was chosen to select solutions, which present a compromise on high CRC values, but still with acceptably low RMSD. Optimization was performed by systematically varying the number of iterations, level of filtration and toggling the use of point spread function. The search for harmonized reconstruction parameters was not an optimization process in the standard sense, but rather a simple but exhaustive evaluation over all possible combinations of image reconstruction parameters. Our harmonization methodology follows the technique proposed Byrd et al. [12], Sunderland et al. [7] and Makris et al. [11] which consists at search through all available combinations of the number of iterations and level of smoothing and determining the parameter sets that best match in contrast recovery curves across small spheres [7, 11, 12].

The analysis was performed using the entire 30 min of data first, and in a second step, by averaging the results of the six independent realizations of approximately 5 min. Five minutes of listmode data more closely simulates the statistics of a clinical scan oncology FDG, however, leading to larger ensemble noise. An alternate harmonization methodology is presented in Supplemental data where 6 sets of noise realizations of 5 min for each image reconstruction parameter sets are employed to identify harmonized reconstruction parameters. CRC curves agreement was generated for mean, max and peak CRC.

## Results

On the mMR, the phantom preparation resulted in average activity concentrations of 1767 Bq/mL ± 5.0% and 17,053 Bq/mL ± 6.0% for the background volume and the spheres, respectively, at imaging times. These activity concentrations correspond to an average ratio of 9.76 ± 0.10. For the GE Signa, these values were 1622 Bq/mL ± 3.4% and 15,526 Bq/mL ± 5.1%, corresponding to an average ratio of 9.57 ± 0.22. The average water volume of the phantom as determined by weight was 9737 ± 11 mL. Sorting the listmode data into six realizations of approximately 5 min resulted in an average of 47.3 ± 0.18 million trues per realization for the GE Signa and 42.0 ± 0.08 million trues per realization for the mMR.

**Fig. 2:**
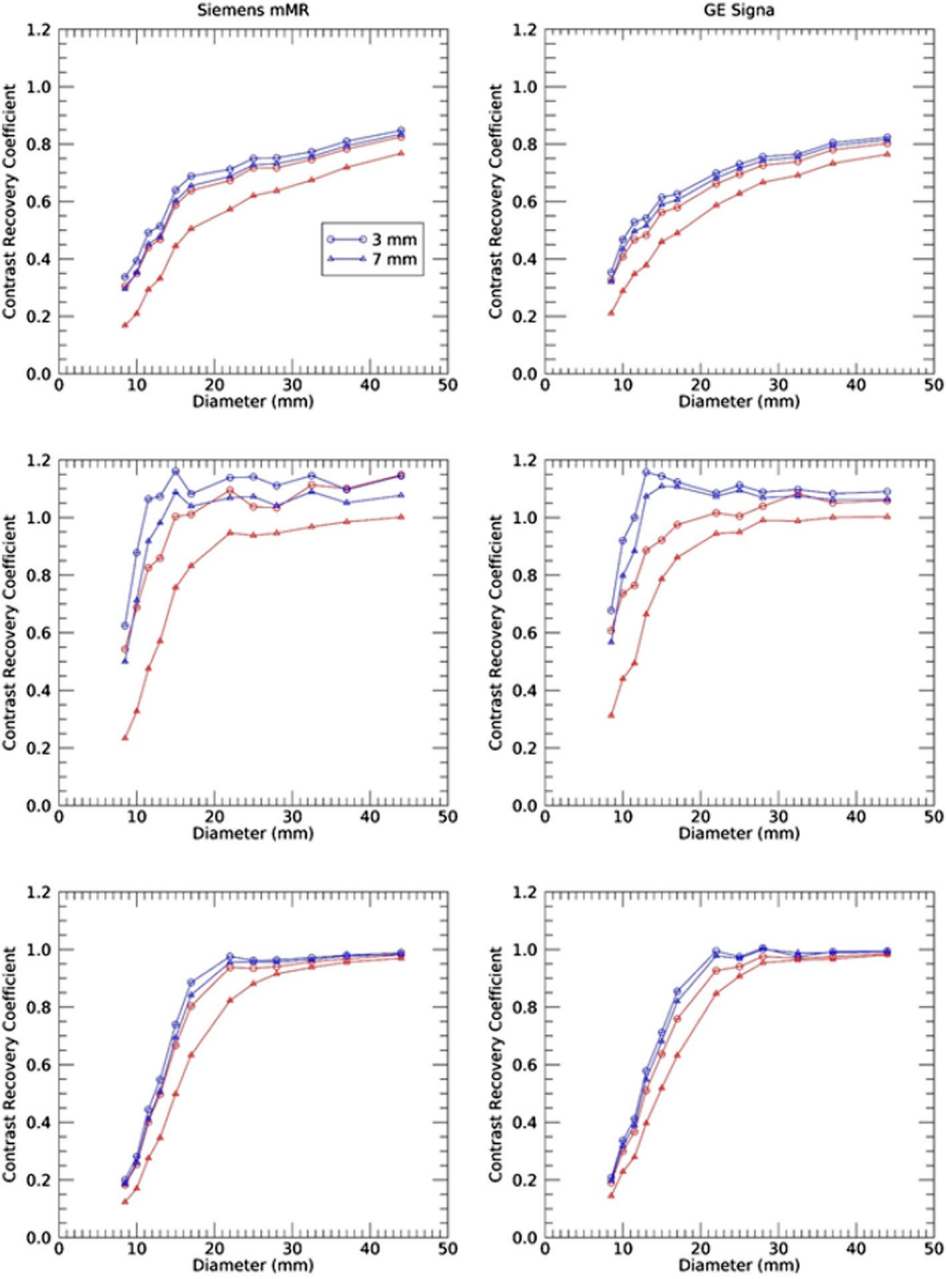
Contrast recovery coefficient (CRC) curves for mean (top), maximum (middle) and peak (bottom), without resolution recovery, either PSF or IR (red), and with resolution recovery (blue) for the GE Signa (right) and Siemens mMR (left) scanners. CRC curves are presented for two post-reconstruction filter widths. Only the least filtration (3 mm) and highest filtration (7 mm) are shown

The range of clinically relevant image reconstruction parameters employed demonstrated widely different quantitative performances across the two manufacturers with regard to recovery of activity measurement as a function of object size. By varying both the number of iterations and post-reconstruction filter level, bands of CRC curves were obtained that showed significant overlap between the two PET/MRI scanners. Contrast recovery coefficient curves for both systems using the full 30 min of acquisition data and four iterations and post-reconstruction filters of 3 and 7 mm are shown in Fig. 2. (Only these two filters are presented for clarity.) CRC curves are presented for mean, max and peak hereafter referred to as CRC_Mean_, CRC_Max_ and CRC_peak_. These plots depict the range of CRC values, and thus size-dependent SUV values, that are obtained in the clinical setting by varying the post-reconstruction filter and using the resolution recovery algorithm option. In this figure, the effect of increasing post-reconstruction filtration (from 3 to 7 mm) is illustrated; as expected, less filtration consistently led to higher CRC values. The largest effect of the resolution recovery algorithm is observed on the CRC_max_, with smaller consequences associated with CRC_mean_ and CRC_peak_. Of note, the CRC_peak_ for spheres with diameter less than 13 mm would typically not be defined as these spheres have a volume less than one cm^3^. The CRC_peak_ for these smaller spheres is thus less than that their CRC_mean_ as the VOI_peak_ includes background surrounding activity. These are nevertheless included for comparison and completeness of the present study.

**Fig. 3:**
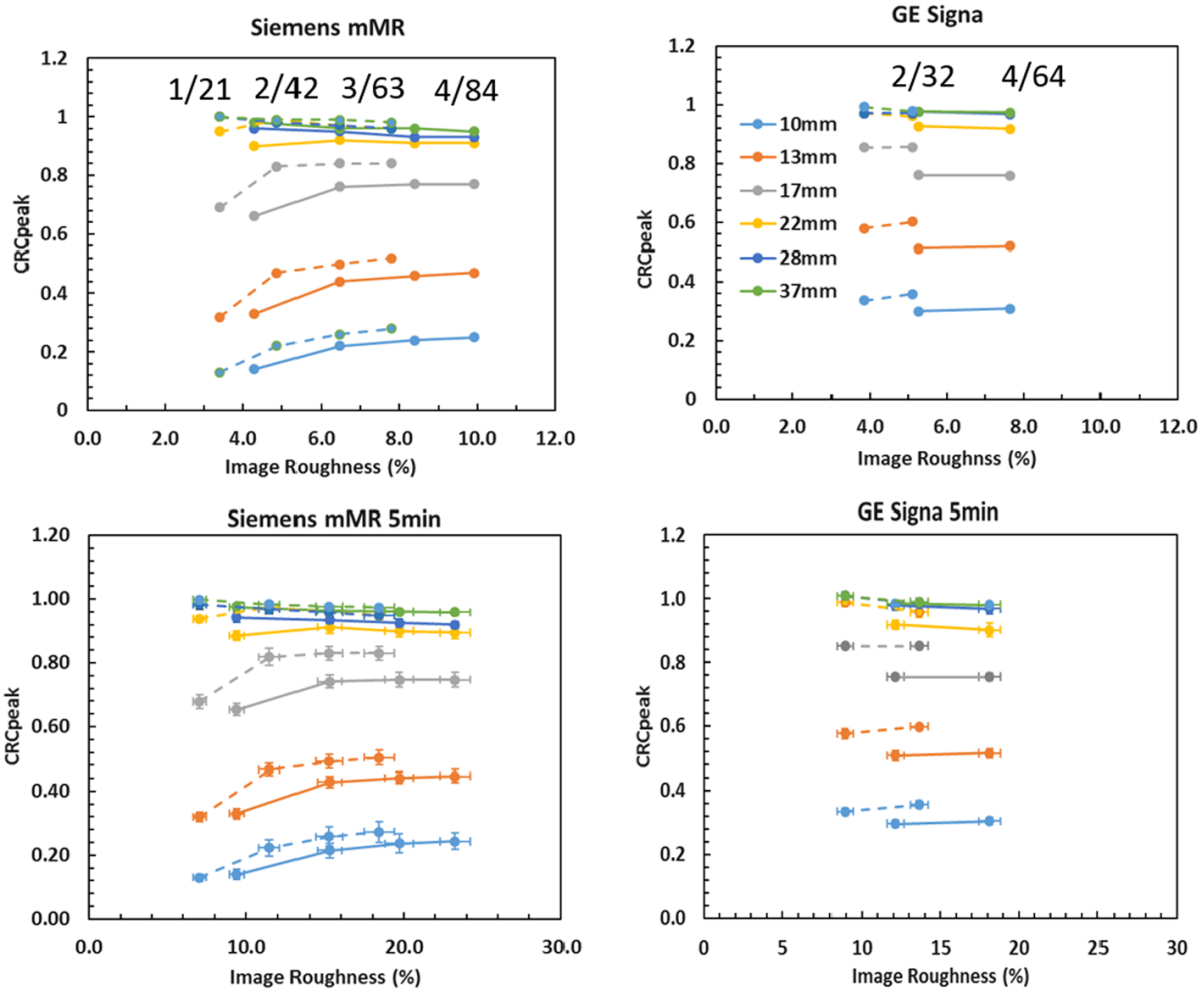
Image roughness versus CRC_peak_ plots for the NEMA standard set of spheres for the Siemens mMR (left) and GE Signa (right). The top row shows plots for the whole 30 min of listmode data and the bottom row, the average value over six frames of 5 min duration. Error bars in the bottom plot show the ensemble noise over the six realizations. The dotted lines represent the reconstructions with the use of resolution recovery (either PSF or IR, depending of vendor). The numbers of iterations and updates are indicated on the top plots

Plots of the image roughness vs CRC_peak_ are presented in Fig. 3 for each scanner for the 30-min and 5-min scans and for the NEMA phantom with the standard set of spheres. Only the 3 mm filtration and CRC_peak_ are presented for brevity. Plots of CRC_mean_ and CRC_max_ are similar (not shown). The filter is applied post-reconstruction and will only contribute to decrease the values of CRC_peak_ and image roughness (reduce noise). On the mMR, as the number of iteration increases from 1 to 4, the CRC_peak_ values increase and reach a maximum value, while the COV (image roughness) increases. This is especially seen on the smallest spheres. For the largest spheres, the CRC_peak_ value is constant for all iteration number and is consistent with the fact that iterative reconstruction converges faster for larger objects. The PSF reconstructions, represented by the dashed lines, show higher CRC and lower image roughness (noise) at the same number of iterations. This is also consistent with PSF which improves resolution and decreases image noise. For the largest spheres, ‘convergence’ is approached as early as 2 iterations, but for the spheres of 17 mm and smaller, the CRC_peak_ values tend to increase slowly indicating more iterations are required for convergence. The image roughness values on the GE Signa datasets are of the same magnitude as for the Siemens mMR, indicating comparable signal/noise characteristics of images from the Signa datasets as compared to the mMR. Slightly lower image roughness is observed on the Signa possibly due to the use of TOF. The CRC_peak_ values at 2 and 4 iterations are approximately identical indicating close to convergence even for the smallest spheres. These observations are consistent with the use of TOF which is known to increase convergence rate and lower noise. The plots for the 5 min reconstructions of image roughness versus CRC_peak_ are presented in the bottom row. The error bars are calculated from the standard deviation over the six noise realizations and correspond to the ensemble noise. We can note that the standard deviation (as denoted by the error bars) over the six noise realizations is less on the GE Signa than on the mMR which is likely due to the use of TOF on GE scanner. The same observations can be made, as for the 30 min datasets, and show that the signal and image noise are highly comparable between the experiments performed on each scanner and that convergence is approached similarly. We observed on this figure that the image roughness is similar, but not the same, and overlaps in magnitude for the phantom prepared in almost identical conditions. We believe this closely represents a situation where a patient similarly prepared would be imaged on the two different scanners and would likely represent that the image roughness will be slightly different for patients imaged on these two systems.

**Fig. 4:**
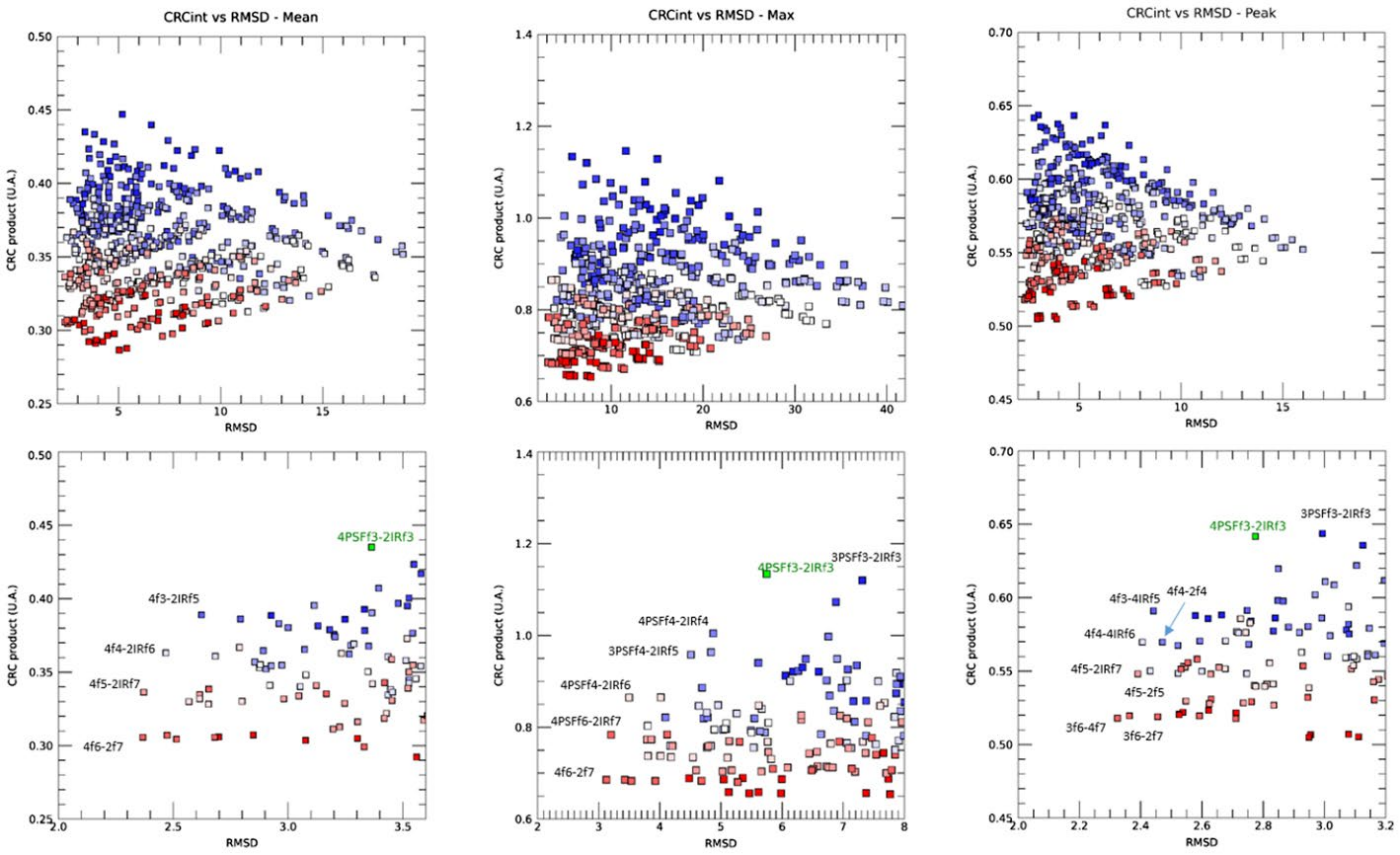
CRC_mean,max,peak_ product versus RMSD box plot. Each of the 600 pairs of image reconstructions parameters combinations is represented by a box (top row). The color scale from blue to red represents the different level of overall filtration. Dark blue boxes represent combinations with least overall filtration (i.e., 3 mm for both mMR and Signa images), and dark red corresponds to most filtration (7 mm on mMR and Signa images). Bottom row, expanded view at low RMSD to identify candidate image reconstruction parameters for optimization. The solution for best RMSD and CRC is indicated in green

Figure 4 presents the distribution the RMSD vs the sum CRC product for all 600 image reconstruction pairs plotted for CRC_mean_, CRC_max_ and CRC_peak_ (top row). In these plots, the sum product of the CRC values is plotted as a function of RMSD for all parameter pairs. In this respect, points on the right side of the plot indicate reconstructions with low filtration and low agreement (high RMSD), points on the bottom left indicate solutions with high agreement (low RMSD) of CRC curves but low resolution (low sum CRC), and solutions on the upper left indicate highest resolution but still with high degree of agreement according to RMSD. We can see that a range of solutions is likely to meet the criteria of harmonization, i.e., low RMSD values or good agreement in CRC curves but with various levels of image resolutions (high CRC product values). In general, solutions with best match in CRC coefficients will thus have a low RMSD and solutions with higher recovery coefficients will have a high CRC_product_. On these graphs, each reconstruction pair is represented by a box whose color depicts the overall amount of filtration, ranging from dark blue (with minimal filtration—both 3 mm on mMR and Signa) to red (maximal filtration, both 7 mm on mMR and Signa), with a gradual progression from blue to red for intermediate levels of filtration. The reconstruction on the mMR with 1 iteration was omitted for clarity as they were shown to have not converged in Fig. 3. The bottom row presents the same plots but with an expanded scale on RMSD with labels for specific optimal reconstruction sets.

Solutions on the left side of these plots are all achieved with low RMSD (good agreement of CRC curves) and varied level of resolution. This indicates multiple optimal solutions exist depending on the imaging task. Setting *β* to 0 leads to the solutions with lowest RMSD. The solutions with the lowest numerical value of RMSD are obtained at the highest filtration of 6 and 7 mm; however, these also correspond to solutions with low sum CRC product. Indeed, optimization by RMSD alone will tend to select solutions with low resolution and reflect more the influence of the post-reconstruction filter rather than the scanner performance. By setting the parameter *β* to ~ 1.0, the solution that minimizes Eq. 4 is represented by the green symbol. These plots show that a common optimized image reconstruction pair exists at 4 iterations with PSF and 3 mm filter for mMR and 2 iterations with IR and 3 mm filter on Signa. This solution corresponds to a high CRC product and an acceptable RMSD value and shows that harmonization solution exists in which both high resolution and excellent agreement in CRC curves can be achieved. The RMSD values using the CRC_peak_ are also, on average, smaller than the RMSD values of CRC_mean_ and CRC_max_.

**Fig. 5:**
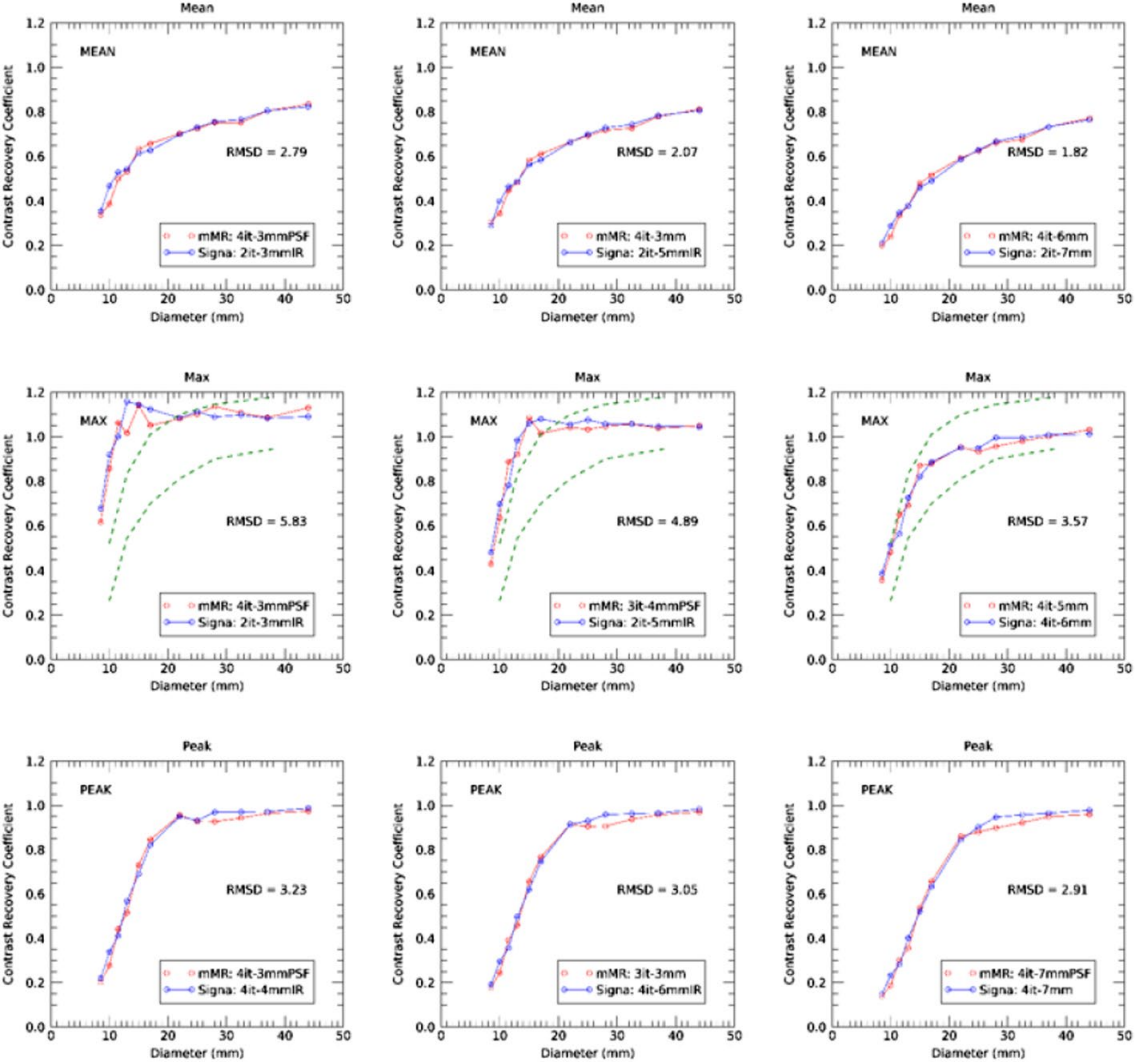
Matching CRC curves for mean (top), max (middle) and peak values (bottom) for 30 min of data corresponding to three optimization scenario: (left) optimized for best resolution or highest CRC values, (middle) optimized for both resolution and RMSD and (right) for best RMSD. The corresponding RMSD values are indicated in each panel. Results from the Siemens Biograph mMR are in red and from the GE Signa are in blue. The dashed lines represent the EANM limits on CRC_max_

Figure 5 presents the CRC curves for the reconstruction parameter set that provide the best match for CRC_mean_, CRC_max_ and CRC_peak_ using the full 30 min of acquired data to provide either lowest RMSD (smoother images—right), (high resolution—left) and an intermediate solution (middle). The solutions with highest sum CRC and low RMSD provide a simultaneous optimization of resolution and CRC agreement. The optimized solution for CRC_max_ shows the highest level of noise, and thus, we provide an intermediate solution with slightly increased filtering as a compromise solution in which excellent agreement in CRC curves is achieved at less noise but still with a low RMSD. The corresponding image reconstruction parameters pairs are reported in Table 2. Excellent agreement between the scanners is observed for the three scenarios, with only subtle differences. At best RMSD match, 100% of recovery (CRC = 1.0) is obtained generally for CRC_max_ or CRC_peak_ for spheres larger than 20 mm diameter and generally for lower CRC values. The RMSD values for three harmonization strategies are reported in Table 2 and Fig. 5. Best match in CRC_mean_ is obtained at 2 iterations/7 mm filter on the GE Signa and 4 iterations/6 mm filter on the Siemens mMR without PSF or IR (RMSD = 1.82). Employing PSF or IR still produces a good agreement of CRC value but at a slight cost of RMSD (RMSD = 2.19), see Additional file 1: Fig. S4. Additional file 1: Fig. S5 presents images of the phantom obtained at reconstruction parameters for best match in CRC_mean_ obtained without and with PSF. It is interesting to note that lower RMSD values are observed for CRC_peak_, than by CRC_mean_ and finally CRC_max_, suggesting that closer ‘harmonization’ can be achieved using SUV_peak_ rather than by SUV_max_ or SUV_mean_. In this figure, the green dashed lines represent the EANM suggested limits in CRC_max_ values for qualification of PET/CT where the recovery coefficients (RCs) values of [24, 25] were converted to CRC by using a known ratio of 10.

**Table 2:** Image reconstruction parameters leading to three optimization criteria for CRC_mean_, CRC_max_ and CRC_peak_ for 30 min data acquisition. All Siemens mMR reconstructions were done with 21 subsets, while all GE Signa reconstructions were done with 16 subsets and TOF (time of flight)

Metric		Optimized high CRC	Intermediate	Lowest RMSD
CRC-Mean	Siemens mMR	4 it – 3 mm + PSF	4 it – 3 mm	4 it – 6 mm
	GE Signa	2 it – 3 mm + IR	2 it – 5 mm + IR	2 it – 7 mm
RMSD		2.79	2.07	1.82
CRC- Max	Siemens mMR	4 it – 3 mm + PSF	3 it – 4 mm + PSF	4 it – 5 mm
	GE Signa	2 it – 3 mm + IR	2 it – 5 mm + IR	4 it – 6 mm
RMSD		5.83	4.89	3.57
CRC-Peak	Siemens mMR	4 it – 3 mm + PSF	3 it – 3 mm	4 it – 7 mm + PSF
	GE Signa	4 it – 4 mm + IR	4 it – 6 mm + IR	4 it – 7 mm
RMSD		3.23	3.05	2.91

### Effect of scan time on harmonized parameters

**Fig. 6:**
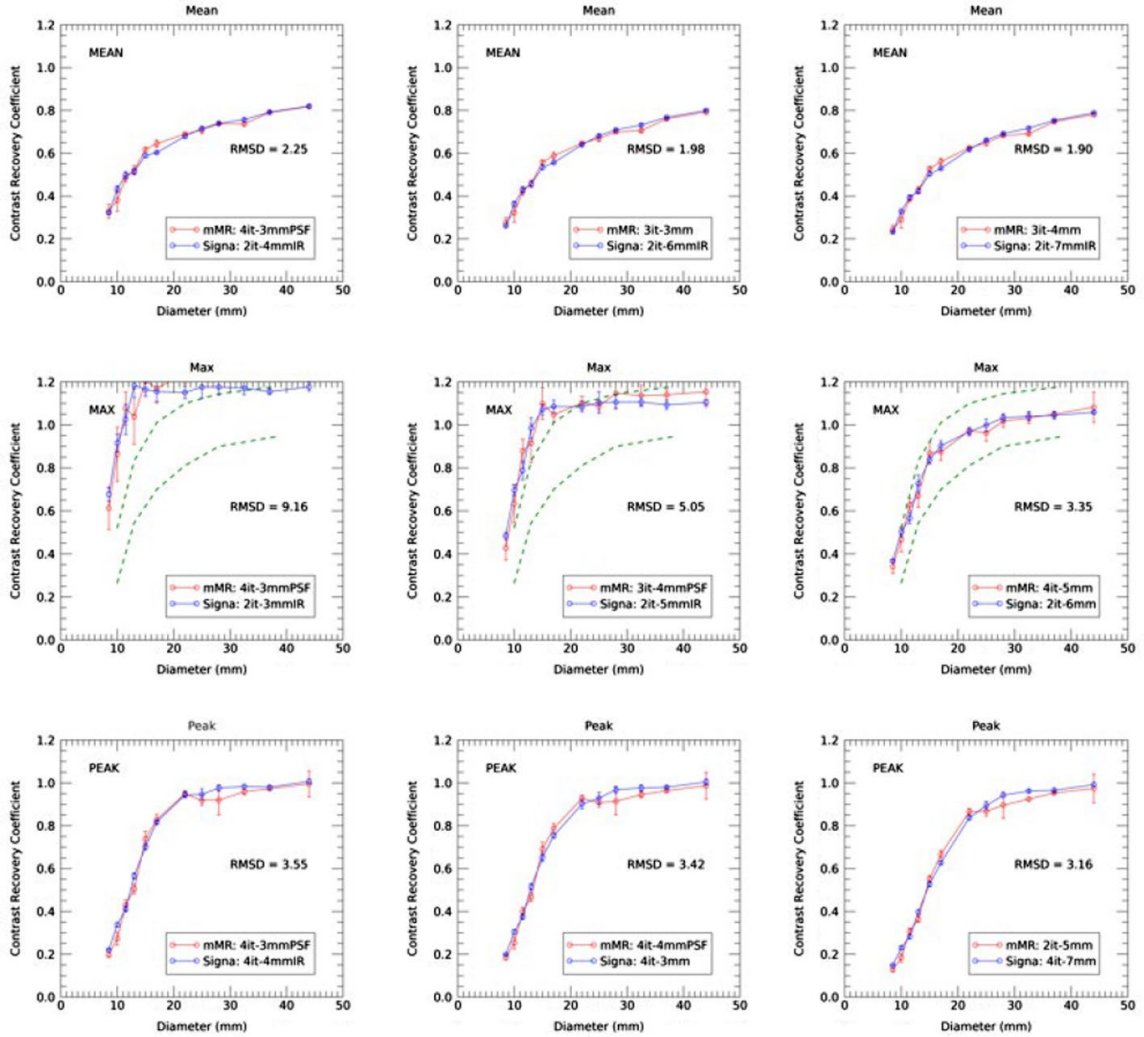
Matching CRC curves for mean (top), max (middle) and peak values (bottom) for 5 min of data corresponding to three optimization scenario: (left) optimized for best resolution or highest CRC values, (middle) optimized for both resolution and RMSD and (right) for best RMSD. The corresponding RMSD values are indicated in each panel. Results from the Siemens Biograph mMR are in red and from the GE Signa are in blue. The dashed lines represent the EANM limits on CRC max

Figure 6 shows the harmonized CRC curves for mean, max and peak values using only 5 min of listmode data. Average values were derived from the six noise realizations. Error bars on these plots correspond to the ensemble noise on the CRC values over the 6 noise realizations. Five minutes of listmode data corresponds to a more clinically relevant scenario as it more closely mimics the data acquisition and the level of statistics encountered in oncology FDG PET/CT scans. Table 3 contains the harmonized image reconstruction parameters obtained using 5 min of listmode data along the three optimization criteria. As in Fig. 5, excellent agreement between the two scanners is found, and similarly harmonized image reconstruction parameters can be determined. The RMSD values are approximately equal to those of  the 30 min acquisition.

**Table 3:** Image reconstruction parameters leading to the three optimization criteria CRC_mean_, CRC_max_ and CRC_peak_ for 5 min data acquisition. All Siemens mMR reconstructions were done with 21 subsets, while all GE Signa reconstructions were done with 16 subsets and TOF (time of flight)

Metric		Optimized high CRC	Intermediate	Lowest RMSD
CRC-Mean	Siemens mMR	4 it – 3 mm + PSF	3 it – 3 mm	3 it – 4 mm
	GE Signa	2 it – 4 mm + IR	2 it – 6 mm IR	2 it – 7 mm + IR
RMSD		2.25	1.98	1.90
CRC- Max	Siemens mMR	4 it – 3 mm + PSF	3 it – 4 mm + PSF	4 it – 5 mm
	GE Signa	2 it – 3 mm + IR	2 it – 5 mm + IR	2 it – 6 mm
RMSD		9.16	4.05	3.35
CRC-Peak	Siemens mMR	4 it – 3 mm + PSF	4 it – 4 mm + PSF	2 it – 5 mm
	GE Signa	4 it – 4 mm + IR	4 it – 3 mm	4 it – 7 mm
RMSD		3.55	3.42	3.16

Figure 6 highlights the high variability in CRC_max_ value when PSF is employed. However, as will be further discussed below, performing harmonization with 5 min listmode data is possible but leads to increased ensemble noise. We used the full 30 min listmode acquisition to minimize the ensemble noise. As noted in Discussion, we believe the largest source of uncertainty is variations in filling the phantom.

CRC curves of images reconstructed using only 5 min of listmode data were compared, employing the image reconstruction parameters that provided the best match from the 30 min scans (data shown in Additional file 1: Fig. S2). Very good agreement in CRC_mean_ was observed for 5 min acquisition using the 30 min harmonization parameters as reflected in the CRC curves and in the value of RMSD. The best agreement for CRC_max_ was found when selecting the 30-min harmonized reconstruction parameters for the intermediate and best RMSD optimization, while the worst agreement is observed for best CRC_max_. The mean and peak CRC curves show generally better agreement than the CRC_max_ curves. The largest differences were observed when looking at CRC_max_ for best 30-min harmonized reconstruction parameters at the high CRC values which reflect the highest noise encountered in the 5-min data. However, in all cases, the overall RMSD values are acceptably small.

The harmonization strategy consisting in using the 6-short 5-min noise realization was performed to identify parameters that minimizes RMSD (for best agreement) which is presented in Additional file 1: Fig. S3. The harmonized parameters were found to be similar to those obtained using the entire 30-min listmode data.

## Discussion

In this study, we determined ‘harmonized’ image reconstruction parameters for CRC_mean_, CRC_max_ and CRC_peak_ for the Siemens mMR and GE Signa PET/MRI systems. The experiments were performed in a controlled setting to focus on variability caused by scanner hardware design and image reconstruction settings. This work excludes errors in measurements due to subjective manual regions of interest definition. CRC variability under clinically relevant range of image reconstruction parameters (iterative updates, Gaussian filtration) and algorithm implementation (3D OSEM, 3D OSEM plus resolution recovery) by each vendor was systematically varied. The imaging protocol was designed and executed to minimize the variability in the phantom preparation by using a rigorous phantom filling procedure, phantom alignment and imaging protocol. In this respect, harmonization objectives differ from other works that aimed at studying the contract recovery reproducibility and algorithm convergence under scan of short duration when ensemble noise is a determinant factor [4, 26, 27]. The key aspect for harmonization in our study is that the phantom be prepared and imaged on the different imaging systems in an identical way and image reconstructions parameters be determined as those that achieve the same level of accuracy across scanners. The image roughness is not expected to be the same as the scanners are physically different and the image reconstruction algorithm implementation is also different. However, we note in Fig. 3 that similar image roughness is achieved with the phantom study indicating the two scanners considered here are not widely different.

The reconstructions were performed using an attenuation map template of the phantom. Since the effect of attenuation correction is decoupled from the choice of reconstruction parameters, our work establishes image reconstruction parameters across the two systems that will allow the study of the consequences in quantitation regarding the accuracy of the recovery coefficients of lesions due to the choice of attenuation correction strategy, as well as subtle implementation differences among the two vendors. Once the image reconstruction parameters are harmonized from the PET data, effects such as choice of attenuation correction, positioning aid and others can be more accurately studied for a given scanner and across scanner vendors. Ultimately, complete harmonization of simultaneous PET/MR scanners will need to include an attenuation correction as measured from the scanner.

Our harmonization methodology that consists in the minimization of the root-mean-square difference follows the work of Sunderland et al. [7] which proposed the summed absolute difference, Makris et al. [11] using the ‘Largest Difference Between Reconstructions’ and Byrd et al. [12] using the ‘Normalized root-mean-square difference.’ In all approaches, the optimal solution is achieved by a systematic search performed by varying the number of iterations, level of smoothing and algorithm options (use of point spread function—PSF, or time of flight—TOF).

This study aimed at identifying harmonized image reconstruction parameters for the two most widely used simultaneous PET/MRI scanners with a multi-fill well-controlled experiment and differs from a multi-site phantom study. Measured variability of quantitative performance between sites using the same make and model scanners comes from two main sources. The first, and most major, is variability of phantom fill. We minimized this variability through performance of rigorous filling procedure, identical at each site. In this study, we minimized the variability by use of long scans and use of identical fill activities, all activities were measured in dose calibrators calibrated to a NIST traceable 511 keV source, and weights were used to access phantom fill volumes.

The second source of error is associated with fundamental intrinsic quantitative performance differences between studies performed on two physically different, but same make and model scanner. These differences, as manifested in the scanner model-specific performance CRC curve on an appropriately calibrated and tuned scanner, are quite small. In fact, precise CRC performance using the NEMA IQ phantom (the same as used in our studies) is used by vendors as acceptance criteria for scanner installations. This variability is small compared to other sources of error, most significantly fill accuracy and precision.

Remarkably similar quantitative performances were achieved through mutual tuning of reconstruction parameters for both the 30-min low-noise case and the clinically relevant 5-min acquisitions. In addition, the harmonization strategy employing the individual noise realization (Additional file 1: Fig. S3) also led to similar harmonized parameters. We need to note here that six noise realizations is not ideal and more would be needed. However, it was not possible to perform more noise realizations with our data. The clinical implication is that if patients are imaged under technically and biologically controlled conditions, but on different PET/MRI systems, prospectively used harmonized reconstruction parameter sets will result in nearly identical quantitative measurements independent of the system used. This conclusion is independent of lesion size. This aspect has important consequences to multicenter clinical trials where data will be aggregated from different models of PET/MR systems.

It should be noted that the ‘harmonized’ image reconstruction parameters are not necessarily those that would yield to the highest CRC values across all spheres. Indeed, we have identified that a harmonization approach relying solely on the lowest RMSD values leads to solutions of high level of filtering and therefore will correspond to very smooth images. This solution will be detrimental for imaging task of lesion detection albeit providing the good agreement in CRC values. This solution emphasizes more the effect of filtering rather than the performance of the scanner, which is appropriate for some clinical and clinical trial applications, but certainly not all. A solution with high CRC values (but still with acceptably low RMSD) would be typically obtained at larger number of iterations and minimal filtering (as shown), but images would be subject to higher noise levels. In particular, the CRC_max_ reaches values significantly higher than 1.0 for image reconstruction with 3-mm post-reconstruction filters and using resolution recovery. We have shown that harmonization solutions exist for which, depending on the imaging task, being either lesion detection (high CRC) or achieving the same level of quantitation accuracy across sites by minimizing RMSD, excellent agreement in CRC values can be achieved between these two scanners.

In cases where images with high CRC values, or site-specific reconstruction protocols, are preferred, two images can be reconstructed, with the second reconstruction using harmonized parameters for quantitation [9]. Alternatively, the first image can potentially be smoothed to match the resolution and noise characteristics of images reconstructed using harmonized parameters for quantitation [28, 29].

The phantom was prepared under conditions mimicking conditions typically encountered in clinical practice with ^18^F-FDG in PET/CT. Imaging protocols suggest imaging at 60-min post-injection of 370–740 MBq (10–20 mCi) ^18^F-FDG from head to mid-thigh in a series of slightly overlapping bed positions, each with axial field of view of 20–25 cm. Although substantial variability exists in clinical PET/CT, typical acquisition times are of the order of 2–4 min per bed position. Therefore, assuming uniform distribution in an average sized human, a typical injection yields to approximately 5000 Bq/mL (e.g., for 555 MBq (15 mCi) injection administer and imaged 60-min post-injection in a 75-kg patient). In this work, the phantom was prepared with a nominal background activity concentration of ~ 1600–1800 Bq/mL, and thus, the 5-min scan would yield similar count statistics to a clinical ^18^F-FDG acquisition of 2 min per bed position with the 30-min study resulting in 6 times the counts as a typical clinical study. The 30-min acquisition data are used to determine the optimal harmonized parameters in images with minimal noise and thus be able to determine image reconstruction parameters that yield most comparable CRC coefficients free from limitations due to statistical noise.

The average activity concentration at imaging time was less in the experiments performed on the GE Signa by approximately 8%. However, this scanner benefits from a higher sensitivity (21 vs. 15 cps/kBq) relative to the Siemens mMR and thus when accounting for the relative scanner sensitivity, more counts were acquired on GE Signa (~ 12% more). In addition, the GE Signa employs time of flight (TOF), while the Siemens mMR does not. The main advantage of TOF is faster convergence and higher signal to noise. This may explain, at least in part, why the best matching CRC curves are obtained with 2 iterations/16 subsets on the GE Signa scanner as opposed to 4 iterations/21 subsets on the Siemens mMR. We performed our study with 16 subsets on the GE Signa scanner. Twenty-eight subsets are also available to the user. However, what matters in terms on convergence is the product of iterations by the number of subsets defining the number of image updates. And consequently, we have performed image reconstruction on the Siemens mMR with number of iterations varied so that the number of image updates (21, 42, 63 and 84) encompasses the range of image updates done on the GE scanner (32 and 64). We have to note that it is impossible to match exactly the number of image updates on two scanners and this is not the intent of harmonization.

CRC_mean_ and CRC_peak_ appear to be more robust metrics used as the basis for harmonization when comparing quantitative results from PET/MRI scanners than CRC_max_ (and thus SUV_max_), as is expected. This is likely an effect of statistical noise even for 30-min datasets, and this effect is greater for 5-min acquisition times. The image noise (image roughness) depends on a variety of factors in the image reconstruction chain (number of iterations and post-reconstruction filter) and includes the choice of algorithm, use of TOF and especially the use of resolution recovery. As such, image noise cannot be rigorously compared. However, the reconstructed noise was determined by the image roughness in the reconstructed images and for these matched experiments, (identical fill and imaging in similar conditions), comparable signal and noise were achieved between the two cameras. In phantom studies, SUV_mean_ is highly robust since the lesion volume is known and the activity distribution within the lesion is uniform. This is not the case in patient studies, and extreme variability is observed in segmentation volume making it of little clinical use, currently. So lesion SUV_mean_ is not recommended within the context of clinical trial response assessment. SUV_max_ is most typically used. Inter-reader measurement variability of SUV_max_ is small, and it is a robust measurement, although impacted significantly by image noise. SUV_peak_ has slowly been gaining acceptance as a more robust (less sensitive to noise) metric of response, although literature support for its use is less prevalent, currently. Similarly, our data indicate that SUV_peak_ is likely the most repeatable measure among the three and that SUV_max_ being more affected by noise. SUV_peak_ will generate higher SUV values than SUV_mean_; however, it can only be defined for lesion larger than 1 cm. In studies where quantitative harmonization is a critical aspect to the trial’s response assessment, then tighter harmonization appears to be achievable when using the SUV_peak_ metric*.* The SUV_peak_ metric seems more independent on the choice of region of interest and the level of smoothing, thus making this metric more amenable to harmonization. It has been previously reported that SUV_max_ is advantageous with respect to the ease of drawing ROIs and that this metric is less susceptible partial-volume effects [24]. Similarly to SUV_max_, SUV_peak_ can be calculated from a sufficiently large VOI traced on the tumor volume using an automated software. Most commercial image review software vendors such as MIM or HERMES provide SUV_peak_ tools which makes this metric user independent. Our data, in agreement with the work Rahmim and Tang [5] and Tong et al. [4], indicate that SUV_max_ shows largest fluctuations especially when using PSF and also is possibly subject to overshoot, i.e., generating recovery coefficient larger than 1. SUV_max_ is also subject to larger fluctuations, especially in small lesions, due to ensemble noise and is likely less reproducible. In this work, we have attempted to minimize ensemble noise with longer acquisition time than typically employed for clinical scans. Lodge [24] concluded that SUV_max_ is best in situation of high statistics but also indicated that SUV_peak_ is more robust at assessing the most metabolically active region of the tumors. Lodge work refers to a previous generation of PET/CT scanner with lower spatial resolution (6.3 × 4.7 × 30 mm crystals) and did not use PSF. Our work, along with Rahmim and Lodge works, thus agrees and points to the limitations of SUV_max_ robustness especially with newer high-resolution scanners and image reconstruction algorithms incorporating resolution recovery.

A limitation of this study is that the experiments were performed in phantoms at a set count density in the spheres and background activity. Extrapolation to human imaging is not directly translatable as the OSEM algorithm is not linear and performance will be dependent on the specific patient activity distribution and count density. This limitation is common to all studies in phantoms. An alternative approach could be to insert synthetic lesions of varied activity (SUV), size and shapes in clinical patient datasets. This is currently an active area of research that we are investigating.

Our data indicate that the harmonized images reconstruction parameters proposed here (both in the ideal long scanning acquisition and in clinical conditions) for PET/MR scanners can be achieved and that comparable size-dependent recovery coefficients or size-dependent tumor SUV values can be obtained and well within the limits proposed by EARL-EANM. PET data acquired on PET/MR scanners would thus be acceptable to be included in multicenter clinical trials, at least as defined by the EARL-EANM criteria. However, this study goes beyond EARL-EANM as it determines image reconstruction parameters that provide practically identical CRC curves between these two scanners and thereby show that variability of in small lesions quantitation can be largely eliminated by controlling the image reconstruction parameters. This conclusion is important as it will allow to further study other factors affecting quantitative PET in PET/MRI such as the specific choice of attenuation correction technique (including the level at which the bones are included), patient positioning aids and others.

## Conclusion

Quantitative PET is influenced by a variety of technical, biological and physical factors. This work demonstrates that harmonization of reconstruction parameters in PET in simultaneous PET/MR is possible and can yield images with nearly identical quantitative performance in terms of CRC measurements over a range of lesion sizes. For the two commercially available PET/MRI scanners evaluated, user-selectable parameters that control iterative updates, image smoothing and PSF modeling provide a range of contrast recovery curves that allow harmonization. This work demonstrates that essentially identical CRC curves can be obtained on two commercially available scanners by a proper choice of image reconstruction parameters. This work will form the basis of further study on the quantitative performance related to the choice of attenuation correction strategy.

## Supplementary Information

**Additional file 1**. PET/MR Harmonization Supplemental data.

## Data Availability

The datasets used and analyzed during the current study are available from the corresponding author on reasonable request.

## References

[1] Boellaard R (2009). Standards for PET image acquisition and quantitative data analysis. J Nucl Med.

[2] Delbeke D, Coleman RE, Guiberteau MJ (2006). Procedure guideline for tumor imaging with 18F-FDG PET/CT 1.0. J Nucl Med.

[3] Graham MM, Wahl RL, Hoffman JM (2015). Summary of the UPICT Protocol for 18F-FDG PET/CT imaging in oncology clinical trials. J Nucl Med.

[4] Tong S, Alessio AM, Kinahan PE (2010). Noise and signal properties in PSF-based fully 3D PET image reconstruction: an experimental evaluation. Phys Med Biol.

[5] Rahmim A, Tang J (2013). Noise propagation in resolution modeled PET imaging and its impact on detectability. Phys Med Biol.

[6] Alliance RQIB. QIBA profile. FDG-PET/CT as an imaging biomarker measuring response to cancer therapy. 2013; Available from: http://www.rsna.org/uploadedfiles/rsna/content/science_and_education/qiba/qiba_fdg-pet_profile_v105_publicly_reviewed_version_final_11dec2013.pdf. 10.1148/radiol.2019191882 PMC7053216 31909700

[7] Sunderland J, Kinahan P, Karp J (2015). Development and testing of a formalism to identify harmonized and optimized reconstructions for PET/CT in clinical trials. J Nucl Med.

[8] Boellaard R, Delgado-Bolton R, Oyen WJG (2015). FDG PET/CT: EANM procedure guidelines for tumour imaging: version 20. Eur J Nucl Med Mol Imaging.

[9] Aide N, Lasnon C, Veit-Haibach P (2017). EANM/EARL harmonization strategies in PET quantification: from daily practice to multicentre oncological studies. Eur J Nucl Med Mol Imaging.

[10] Sunderland J, Kinahan P, Karp J (2016). Assessment of PET/CT reconstruction harmonization through Gaussian post- filtration. J Nucl Med.

[11] Makris NE, Huisman MC, Kinahan PE (2013). Evaluation of strategies towards harmonization of FDG PET/CT studies in multicentre trials: comparison of scanner validation phantoms and data analysis procedures. Eur J Nucl Med Mol Imaging.

[12] Byrd D, Sunderland J, Scheuermann J (2014). A post-hoc methodology for harmonizing PET image reconstruction protocols. J Nucl Med.

[13] Delso G, Fürst S, Jakoby B (2011). Performance measurements of the siemens mMR integrated whole-body PET/MR scanner. J Nucl Med.

[14] Grant AM, Deller TW, Khalighi MM (2016). NEMA NU 2–2012 performance studies for the SiPM-based ToF-PET component of the GE SIGNA PET/MR system. Med Phys.

[15] Daube-Witherspoon ME, Karp JS, Casey ME (2002). PET performance measurements using the NEMA NU 2–2001 standard. J Nucl Med.

[16] Boellaard R, O’Doherty MJ, Weber WA (2010). FDG PET and PET/CT: EANM procedure guidelines for tumour PET imaging: version 10. Eur J Nucl Med Mol Imaging.

[17] Jeong JH, Cho IH, Kong EJ (2014). Evaluation of dixon sequence on hybrid PET/MR compared with contrast-enhanced PET/CT for PET-positive lesions. Nucl Med Mol Imaging.

[18] Hudson HM, Larkin RS (1994). Accelerated image reconstruction using ordered subsets of projection data. IEEE Trans Med Imaging.

[19] Vandenberghe S, Mikhaylova E, D'Hoe E (2016). Recent developments in time-of-flight PET. EJNMMI Phys.

[20] Surti S (2015). Update on time-of-flight PET imaging. J Nucl Med.

[21] Ziegler S, Braun H, Ritt P (2013). Systematic evaluation of phantom fluids for simultaneous PET/MR hybrid imaging. J Nucl Med.

[22] Liow J, Strother SC (1991). Practical tradeoffs between noise, quantitation, and number of iterations for maximum likelihood-based reconstructions. IEEE Trans Med Imaging.

[23] Lodge MA, Chaudhry MA, Wahl RL (2012). Noise considerations for PET quantification using maximum and peak standardized uptake value. J Nucl Med.

[24] Boellaard R. EANM FDG PET/CT accrediation specifcations for SUV recovery coefficients. 2019 [cited 2019 5/30/2019]; Available from: http://earl.eanm.org/cms/website.php?id=/en/projects/fdg_pet_ct_accreditation/accreditation_specifications.htm.

[25] Boellaard R, Rausch I, Beyer T (2015). Quality control for quantitative multicenter whole-body PET/MR studies: a NEMA image quality phantom study with three current PET/MR systems. Med Phys.

[26] Rahmim A, Qi J, Sossi V (2013). Resolution modeling in PET imaging: theory, practice, benefits, and pitfalls. Med Phys.

[27] Cheng J-CK, Shoghi K, Laforest R (2012). Quantitative accuracy of MAP reconstruction for dynamic PET imaging in small animals. Med Phys.

[28] Kelly MD, Declerck JM (2011). SUVref: reducing reconstruction-dependent variation in PET SUV. EJNMMI Res.

[29] Jagust WJ, Bandy D, Chen K (2010). The Alzheimer's disease neuroimaging initiative positron emission tomography core. Alzheimers Dement.

