# Supplementary material for: Harmonization of PET image reconstruction parameters in simultaneous PET/MRI
Source: EJNMMI Phys. 2021 Nov 5;8:75. doi: 10.1186/s40658-021-00416-0 (PMC8571452; doi:10.1186/s40658-021-00416-0)
Supplement: Supplementary file 1 — Additional file 1. PET/MR Harmonization Supplemental data. [file 40658_2021_416_MOESM1_ESM.docx]

**PET/MR Harmonization Supplemental data**

Demonstration of registration accuracy of the CT based phantom attenuation template to the PET image reconstructed with no attenuation correction (NAC). The registered template were then scales to PET attenuation coefficient and used for attenuation correction of the PET data.


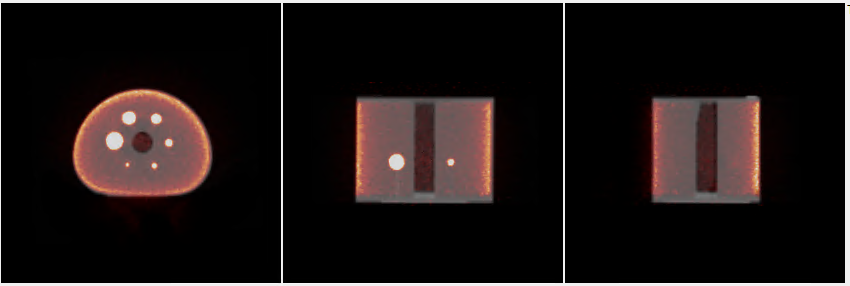


Figure S1. Registration of the CT based phantom attention template to the PET -NAC data.

We compared CRC curves at the image reconstruction parameters leading to best match at 30 minutes from above, but for images reconstructed using only 5 minutes of the listmode data (Figure S2 supplemental data). These images are subject to more statistical noise and are more representative of statistics observed in clinical studies. The top row of this figure shows the CRC curves at mean, maximum and peak values for 5 minutes image reconstructions for the best harmonized CRC_mean_ match obtained at 30min, the middle row is for the best match at 30min on CRC_max_, and finally the bottom row for the best match in CRC_peak_.


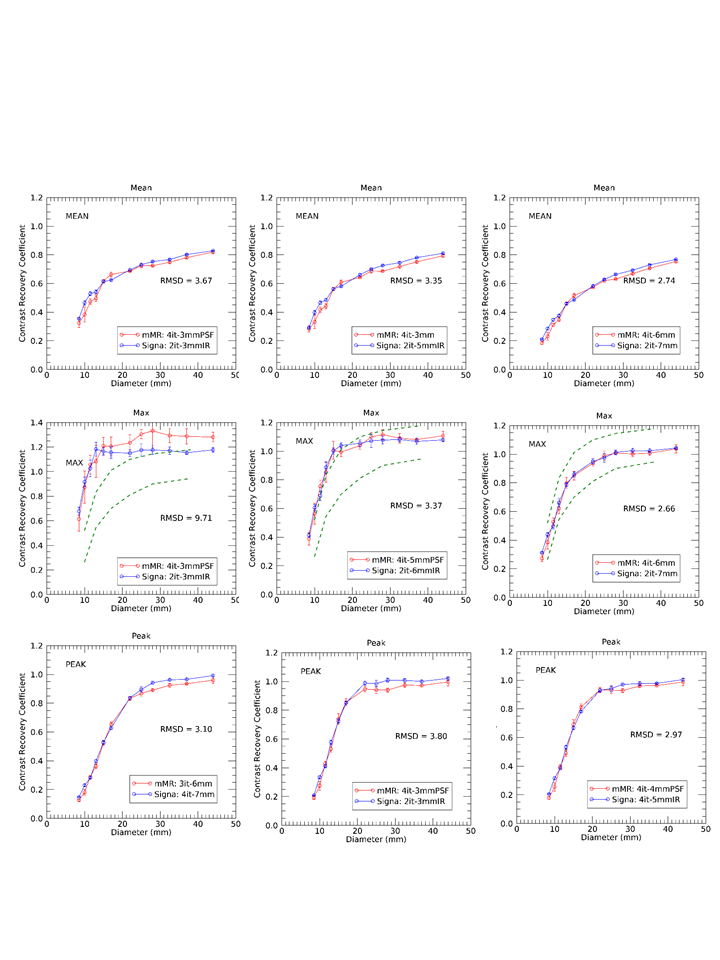


S2. CRC curves at 5 minutes for (top) best CRC_mean_ match using harmonized image reconstruction parameters at 30minutes, (middle) best CRC_max_ match using harmonized image reconstruction parameter at 30minutes, and (bottom) best CRC_Peak_ match using harmonized image reconstruction parameter at 30minutes.

Harmonization methodology using noise realizations

Harmonization was also performed using multiple short realizations. Here, we used the six, 5 minutes realizations from each scanner and each image reconstruction hyperparameter sets. For a given hyper-parameter pair, there are thus 36 frame-to-frame permutations, and thus for each hyperparameter pair, the mean and standard deviation on RMSD and CRC_product_ were computed. Those values are represented in the CRC_product_ vs RMSD plots below as colored symbols with error bars on Figure S2. The plot show the solutions leading to lowest RMSD for CRC_mean_, CRC_max_ and CRC_peak_. The error bars on the figure were determined by the standard deviation on each reconstruction pairs. We observe that error bars on RMSD are rather large (as seen on the narrow horizontal scale shown below) relative to the error bars on CRC_product_. This indicates that the ensemble noise effect is to randomly affect the CRC values which then lead to frame-frame permutation fluctuations in RMSD. However, ensemble noise has little effect of the sum of the CRC values across sphere sizes. This is understandable due to consideration on ensemble image noise in 5 minute acquisition. What we see is that the solutions with lowest RMSD matches approximately the solutions that led to the lowest in RMSD using the 30min listmode data (Table S1 below).

Table S1. List of the top three solutions with lowest RMSD for CRC_mean_, CRC_max_ and CRC_peak_. RMSD values are provided in parenthesis and comparison is made to the harmonized hyperparameters obtained using the entire 30min listmode.

| Harmonized Hyperparameters at 6 x 5minutes | | |
| --- | --- | --- |
| CRC_mean_ | CRC_max_ | CRC_peak_ |
| 4it-5mm 4it-7mm (2.47)  3it-5mm 2it-7mm (2.47)  3it-5mm 4it-7mm (2.48) | 4it-6mm 4it-7mm (5.15)  4it-6mm 2it-7mm (5.16)  4it-7mm+PSF 2it-7mm (5.18) | 4it-7mm+PSF 4it-7mm (4.04)  3it-7mm+PSF 4it-7mm (4.05)  2it-5mm 4it-7mm (4.05) |
| Harmonized Hyperparameters at 30 minutes | | |
| 4-6mm 2it-7mm (1.82) | 4it-5mm 2it-6mm (3.57) | 4it-7mm+PSF 4it-7mm (2.91) |


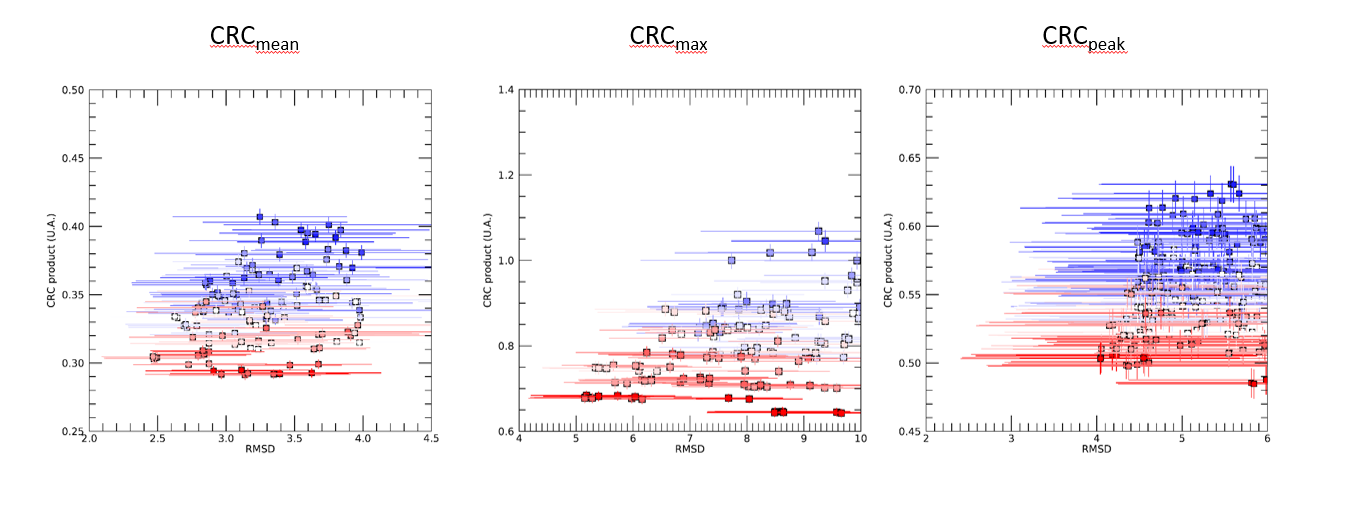


Figure S3. CRC_mean,max,peak_–product vs RMSD box plot. Each of the 600 pairs of image reconstructions hyperparameters combinations is represented by a box using the 6x5min ensemble noise realization. Only combinations leading to RMSD values less than 4.5 (CRC_mean_) 10 (CRC_max_) and 6 (CRC_peak_) are plotted. Error bars represents the standard deviation on the CRC product and RMSD over the 36 frame-to-frame combination for particular hyperparameter set. The color scale from blue to red represents the different level of overall filtration.


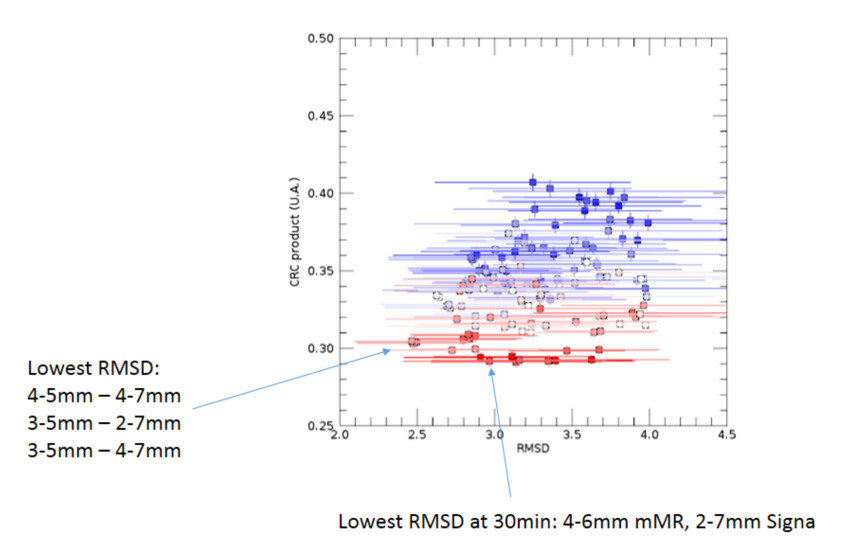
Figure S3 (insert)

CRC_mean_–product with identification of candidate image reconstruction parameters for harmonization.

The Best match in CRC_mean_ is obtained at 2 iterations/7mm filter on the GE Signa and 4 iterations/6mm filter on the Siemens mMR without the use of PSF or IR (RMSD = 1.82). Employing PSF or IR still produces a good agreement of CRC value but at a slight cost of RMSD (RMSD=2.19).


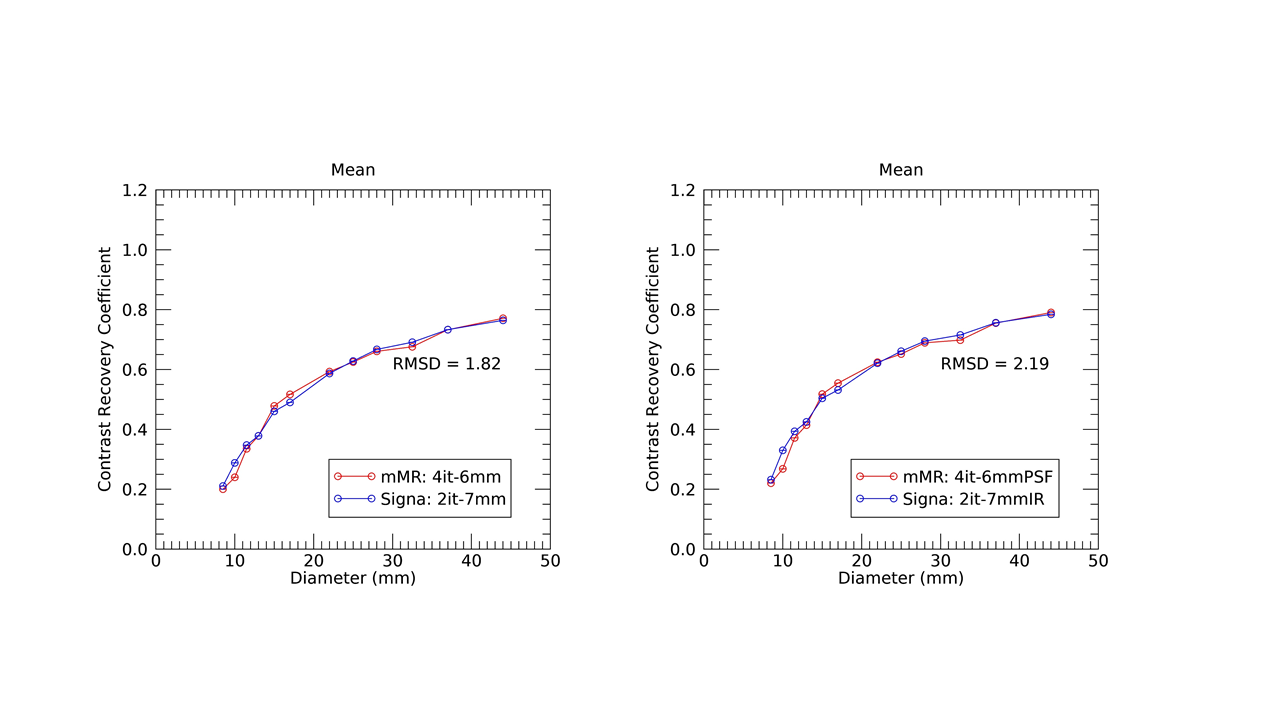


Figure S4. Comparison of CRC curves at lowest RMSD, without and with PSF/IR.


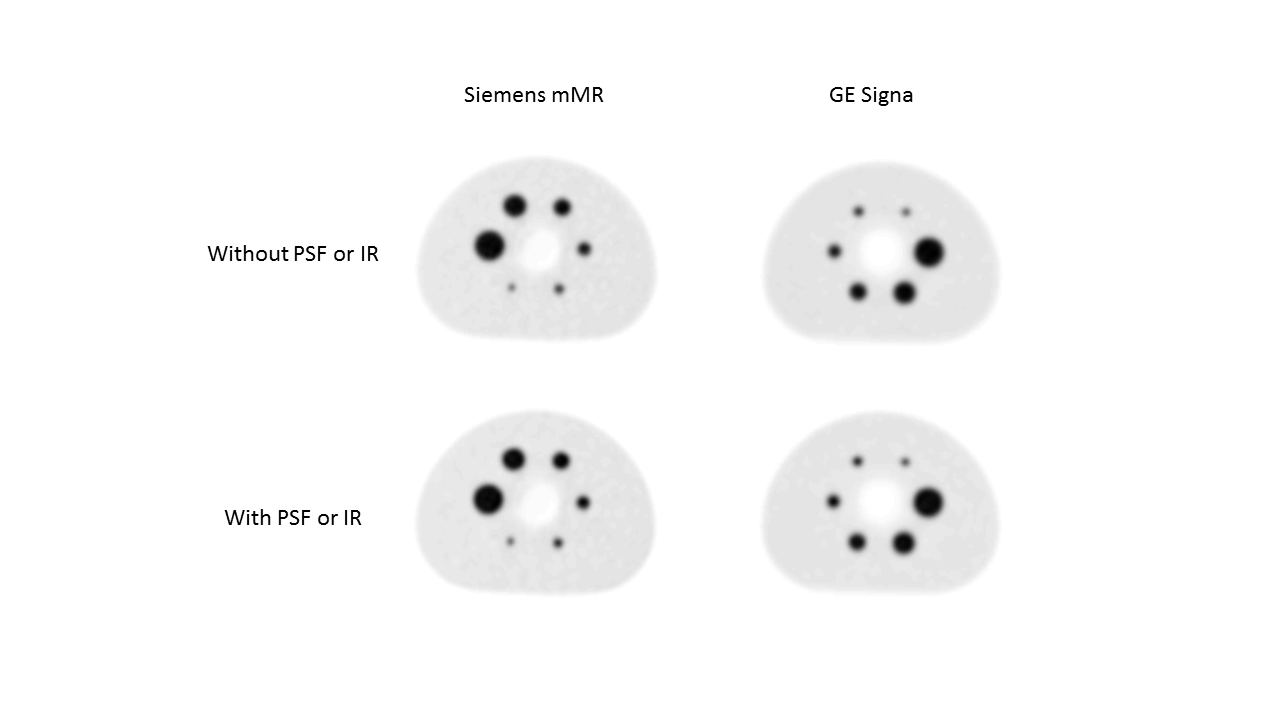


Figure S5. Transverse and coronal images of the phantom obtained with reconstruction parameters at best match in CRC mean without PSF or IR, and with PSF or IR.
